# Manganese Oxide-Functionalized Palm Leaf Biochar for High-Efficiency Ciprofloxacin Adsorption from Water

**DOI:** 10.3390/ijms27188190

**Published:** 2026-09-15

**Authors:** Samah Daffalla, Mohamed R. El-Aassar

**Affiliations:** 1 Department of Environment and Agricultural Natural Resources, College of Agricultural and Food Sciences, King Faisal University, P.O. Box 400, Al-Ahsa 31982, Saudi Arabia; 2 Department of Chemistry, College of Science, Jouf University, P.O. Box 2014, Sakaka 72388, Saudi Arabia

**Keywords:** ciprofloxacin, MnO_2_ nanoparticles, green synthesis, adsorption, antibiotic removal, biochar nanocomposite

## Abstract

The widespread occurrence of pharmaceutical contaminants, especially ciprofloxacin (CIP), in aquatic environments has created an urgent need for efficient and environmentally friendly water treatment strategies. In the present study, a green manganese oxide palm leaf biochar (MnO_2_/PL biochar) nanocomposite was synthesized by depositing MnO_2_ nanoparticles onto palm leaf-derived biochar utilizing *Acacia nilotica* pod extract as an eco-friendly reducing and stabilizing agent. Structural and surface characterization using SEM, EDX, FTIR, XRD, and BET verified the successful synthesis of the composite and revealed a substantial enhancement in its textural properties, leading to an approximately 42-fold increase in specific surface area, from 1.7 to 71 m^2^/g. The MnO_2_/PL biochar exhibited excellent CIP adsorption performance, achieving 95% removal efficiency. The Langmuir model provided the best fit to the equilibrium data, indicating monolayer adsorption behavior, with a maximum adsorption capacity (*Q*_max_) of 24 mg/g. The adsorption kinetics were most accurately described by the pseudo-second-order model (*R*^2^ = 0.999), indicating that CIP uptake was governed by interactions with available active sites on the composite surface. The enhanced performance was attributed to the synergistic effects of the porous biochar structure and MnO_2_ nanoparticles, which promoted multiple adsorption interactions. Furthermore, the regeneration study demonstrated that the MnO_2_/PL biochar retained 81% of its initial CIP removal efficiency after four adsorption–desorption cycles, indicating good reusability and potential for repeated application. The MnO_2_/PL biochar composite shows strong potential as a sustainable adsorbent for antibiotic removal from wastewater.

## 1. Introduction

Pharmaceutical residues are increasingly recognized as emerging contaminants because of their extensive production, widespread consumption, and frequent occurrence in surface water, groundwater, and treated wastewater [1]. Among these contaminants, fluoroquinolones (FQs) constitute one of the most commonly used classes of antibiotics in both human and veterinary medicine. Their persistence in the environment is largely attributed to their resistance to biodegradation over a broad concentration range [1,2]. Among fluoroquinolone antibiotics, ciprofloxacin (CIP) is one of the most extensively prescribed second-generation compounds and is of particular concern due to its high aqueous solubility across a wide pH range, environmental stability, and incomplete metabolism following administration, which contribute to its continuous discharge into aquatic systems [1]. Consequently, CIP is frequently detected in wastewater and natural water bodies, where its accumulation poses significant environmental challenges. The persistent presence of CIP has been associated with the emergence and proliferation of antibiotic-resistant microorganisms, compromising the effectiveness of antimicrobial therapies and threatening both ecological integrity and water quality. Several technologies have been explored for wastewater purification, including biological treatment, membrane filtration, advanced oxidation processes (AOPs), and adsorption [3]. Although biological processes are generally economical, their effectiveness against complex organic pollutants is often limited. Membrane-based technologies can achieve high removal efficiency but suffer from membrane fouling and elevated operational costs. Likewise, AOPs effectively degrade numerous contaminants but may require substantial energy input and can generate secondary by-products. Among these treatment approaches, adsorption has become one of the most widely adopted techniques because of its simple operation, cost-effectiveness, high removal efficiency, and broad applicability for removing a wide range of pollutants [4,5]. Recent studies have shown that CIP adsorption varies considerably among biochars and engineered composite adsorbents because of differences in biomass source, preparation conditions, and surface characteristics. For example, Langmuir adsorption capacities of approximately 21.0 mg/g for banana-peel biochar and 78.4 mg/g for bamboo-sawdust biochar have been reported [2,6]. These variations demonstrate that the adsorption performance of unmodified biochar is strongly dependent on its physicochemical properties. Surface activation and functionalization can improve pore development and introduce additional binding sites, although these approaches may require additional reagents and processing steps [5].

In recent years, nanotechnology has emerged as a promising approach for wastewater treatment, leveraging the distinctive physicochemical properties of nanomaterials, such as their large surface-area-to-volume ratios, superior reactivity, and adjustable surface functionalities. These properties facilitate the effective elimination of various contaminants even at trace concentrations that are difficult to eliminate using conventional treatment methods. Nanomaterials have demonstrated considerable potential in wastewater treatment applications, including adsorption, photocatalytic degradation, and membrane filtration processes, leading to notable improvements in treatment efficiency and contaminant selectivity [7]. Metal oxide modification provides an additional strategy for improving biochar performance because metal–oxygen and surface hydroxyl groups can provide reactive sites for interactions with CIP. In particular, manganese oxide–biochar materials combine the accessible carbonaceous pore structure with the surface reactivity of manganese oxide, potentially providing a broader range of adsorption interactions than pristine biochar [8]. Other multifunctional nanocomposites, such as CoFe_2_O_4_/Mn–Fe LDH-functionalized biochar, can provide multiple adsorption pathways and increased surface functionality; however, their preparation generally involves multiple components and more elaborate synthesis procedures [9]. Thus, although highly engineered nanocomposites can offer enhanced functionality, simpler metal-oxide/biochar systems may provide a practical balance between adsorption performance, material preparation, and sustainability.

Among metal oxides, Manganese oxide (MnO_2_) is particularly attractive because of its surface reactivity and ability to interact with a variety of aqueous contaminants. Green synthesis of MnO_2_ nanoparticles can further improve the environmental compatibility of these materials by reducing the use of conventional chemical reducing or stabilizing agents. Plant-derived compounds can act as natural reducing and capping agents, helping to control nanoparticle formation and aggregation while providing a relatively simple and environmentally benign synthesis route [8]. The use of *Acacia nilotica* pod extract as a green precursor for manganese oxide nanoparticle synthesis has gained attention due to its natural reducing and stabilizing capabilities. The plant, belonging to the family Leguminosae (Fabaceae) and subfamily Mimosoideae, contains diverse phytochemicals, including phenolics, flavonoids, alkaloids, and saponins, which may facilitate the conversion of Mn^2+^ ions and help regulate nanoparticle growth by limiting aggregation [10,11].

Despite the growing number of biochar- and manganese-oxide-based adsorbents investigated for CIP removal, limited attention has been given to combining palm-leaf-derived biochar with green-synthesized MnO_2_ using *Acacia nilotica* pod extract, thereby integrating agricultural waste valorization with an environmentally benign nanoparticle synthesis route. This strategy combines the porous characteristics of palm leaf biochar with the reactive surface properties of MnO_2_ while reducing reliance on conventional chemical synthesis approaches. Accordingly, the present study aimed to synthesize an MnO_2_/palm leaf biochar (MnO_2_/PL biochar) nanocomposite and evaluate its performance for CIP removal from aqueous media through adsorption. Furthermore, the effects of key operational parameters, adsorption kinetics, adsorption isotherms, and regeneration performance over repeated adsorption–desorption cycles were systematically investigated to better understand the adsorption behavior, possible mechanisms, and reusability of the developed nanocomposite.

## 2. Results and Discussion

### 2.1. Characterization of Adsorbents

#### 2.1.1. FTIR Analysis

**Figure 1:**
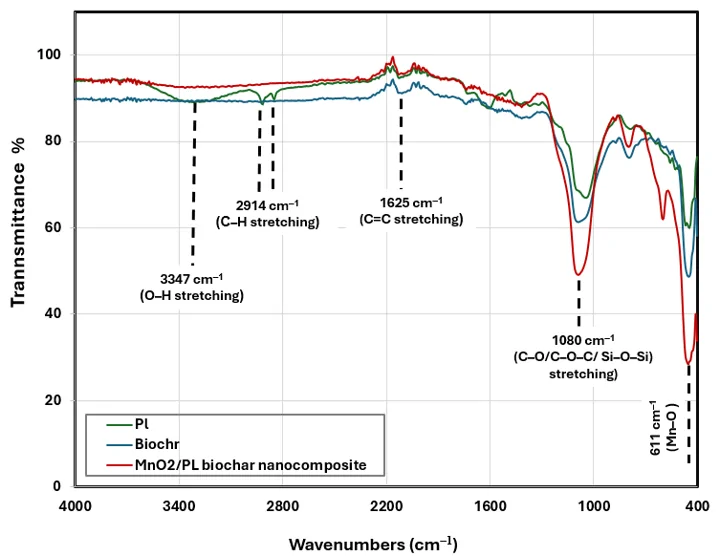
FTIR spectrum of PL, biochar, and MnO_2_/PL biochar nanocomposite.

The surface functional groups and chemical changes associated with the preparation of the MnO_2_/PL biochar nanocomposite were investigated using Fourier Transform Infrared Spectroscopy (FTIR), as shown in Figure 1. The FTIR of PL displayed a broad absorption feature at approximately 3347 cm^−1^, indicating the presence of hydroxyl functionalities from the major lignocellulosic constituents (cellulose, hemicellulose, and lignin). The asymmetric and symmetric stretching modes of aliphatic C–H bonds were identified as the causes of the peaks at 2914 and 2855 cm^−1^, respectively. After pyrolysis at 600 °C, these characteristic peaks became significantly weaker in the PL biochar spectrum, indicating changes in the abundance of oxygen-containing and aliphatic functional groups as a result of thermal treatment and the loss of volatile lignocellulosic components [12]. In addition, the band located around 1625 cm^−1^, commonly attributed to aromatic C=C stretching and oxygen-containing surface functionalities, remained detectable after pyrolysis and MnO_2_ loading, although its intensity decreased relative to that of the raw PL. This variation suggests changes in the chemical environment and relative abundance of surface functional groups following thermal treatment and MnO_2_ incorporation [13]. A band at approximately 1088 cm^−1^ was observed in all spectra and may arise from overlapping C–O/C–O–C stretching vibrations of lignocellulosic structures and Si–O vibrations from mineral components. Similar overlapping contributions have been reported for cellulose/biochar-based materials [14]. Therefore, this band is not assigned exclusively to Si–O–Si, and the possible contribution of silicon-containing phases is further examined by scanning electron microscopy (SEM) coupled with energy-dispersive X-ray spectroscopy (EDX), and X-ray Diffraction (XRD) in the following sections.

For the MnO_2_/PL biochar nanocomposite, the emergence of an absorption band at approximately 611 cm^−1^ provides further evidence for the presence of manganese–oxygen (Mn–O) bonds, which are associated with the deposited manganese oxide phase [15]. Furthermore, slight variations in the position and intensity of the remaining surface functional group bands suggest possible chemical interactions between the manganese oxide phase and the biochar surface during composite preparation.

#### 2.1.2. Morphological and Elemental Analysis (SEM–EDX)

**Figure 2:**
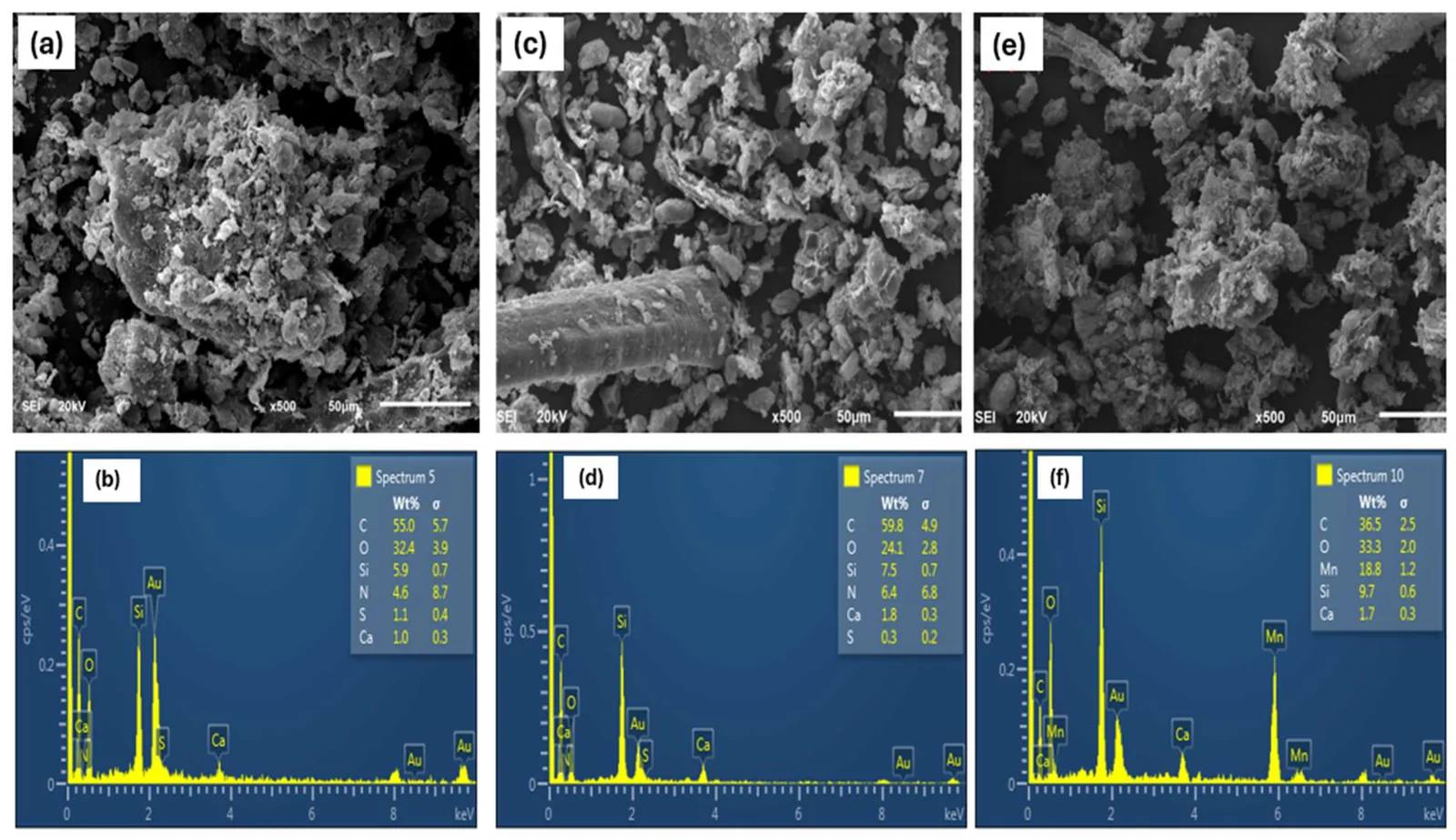
SEM images and corresponding EDX spectra of (**a**,**b**) PL, (**c**,**d**) biochar, and (**e**,**f**) MnO_2_/PL biochar.

The morphological characteristics and elemental profile of the PL sample, pristine biochar, and the MnO_2_/PL biochar nanocomposite were examined using SEM–EDX, as presented in Figure 2. The SEM image of the original PL sample (Figure 2a) showed a compact, smooth, and relatively uniform surface with limited porosity, which is consistent with the structure of untreated lignocellulosic biomass. After pyrolysis at 600 °C, the produced biochar (Figure 2c) displayed a rougher and more irregular morphology, characterized by surface fractures, cavities, and newly developed porous regions. These structural modifications are associated with the thermal degradation of lignin, cellulose, and hemicellulose components, resulting in the elimination of volatile compounds and the formation of a more porous carbon framework [9]. Although the SEM images provide visual evidence of morphological modification, changes in surface area and porosity were evaluated quantitatively using nitrogen adsorption–desorption (BET) analysis rather than inferred from SEM observations alone.

In the MnO_2_/PL biochar nanocomposite (Figure 2e), the surface morphology was further modified, with numerous small bright particles and clusters distributed across the biochar surface. These features are attributed to the deposited manganese oxide phase, while their incorporation is further supported by the Mn signal and its spatial distribution obtained from EDX analysis. The deposition of these particles produced a more heterogeneous and rugged surface morphology, indicating modification of the biochar surface following MnO_2_ incorporation [16].

The elemental analysis performed via EDX confirms the chemical changes associated with each synthesis stage. The EDX spectrum of the raw PL sample (Figure 2b) exhibited predominant signals corresponding to Carbon (C) and Oxygen (O), along with minor peaks for minerals such as Silicon (Si) and Calcium (Ca), which are naturally present in palm waste [17]. The presence of the Gold (Au) peak in all spectra is attributed to the conductive coating applied during sample preparation. For the pristine biochar (Figure 2d), the spectrum reflects a similar elemental profile but with a higher relative carbon content, consistent with the carbonization process. Most importantly, the EDX spectrum of the MnO_2_/PL biochar nanocomposite (Figure 2f) exhibited a distinct Mn signal, with Mn accounting for approximately 18.8 wt% of the detected elements. The appearance of this substantial Mn contribution, together with the corresponding elemental mapping, provides direct compositional evidence for the incorporation of a manganese-containing phase into the biochar matrix [18]. The concurrent presence of Mn and O is consistent with the formation of the MnO_2_ phase, while the spatial distribution of Mn supports its deposition throughout the examined composite region.

Overall, the SEM–EDX results provide complementary morphological, compositional, and spatial evidence for the formation of the MnO_2_/PL biochar composite. Nevertheless, SEM–EDX alone cannot establish the nanoscale structure or quantitatively determine changes in surface area and porosity. These properties are therefore assessed separately using XRD and N_2_ adsorption–desorption/(BET) analysis.

#### 2.1.3. XRD Analysis

**Figure 3:**
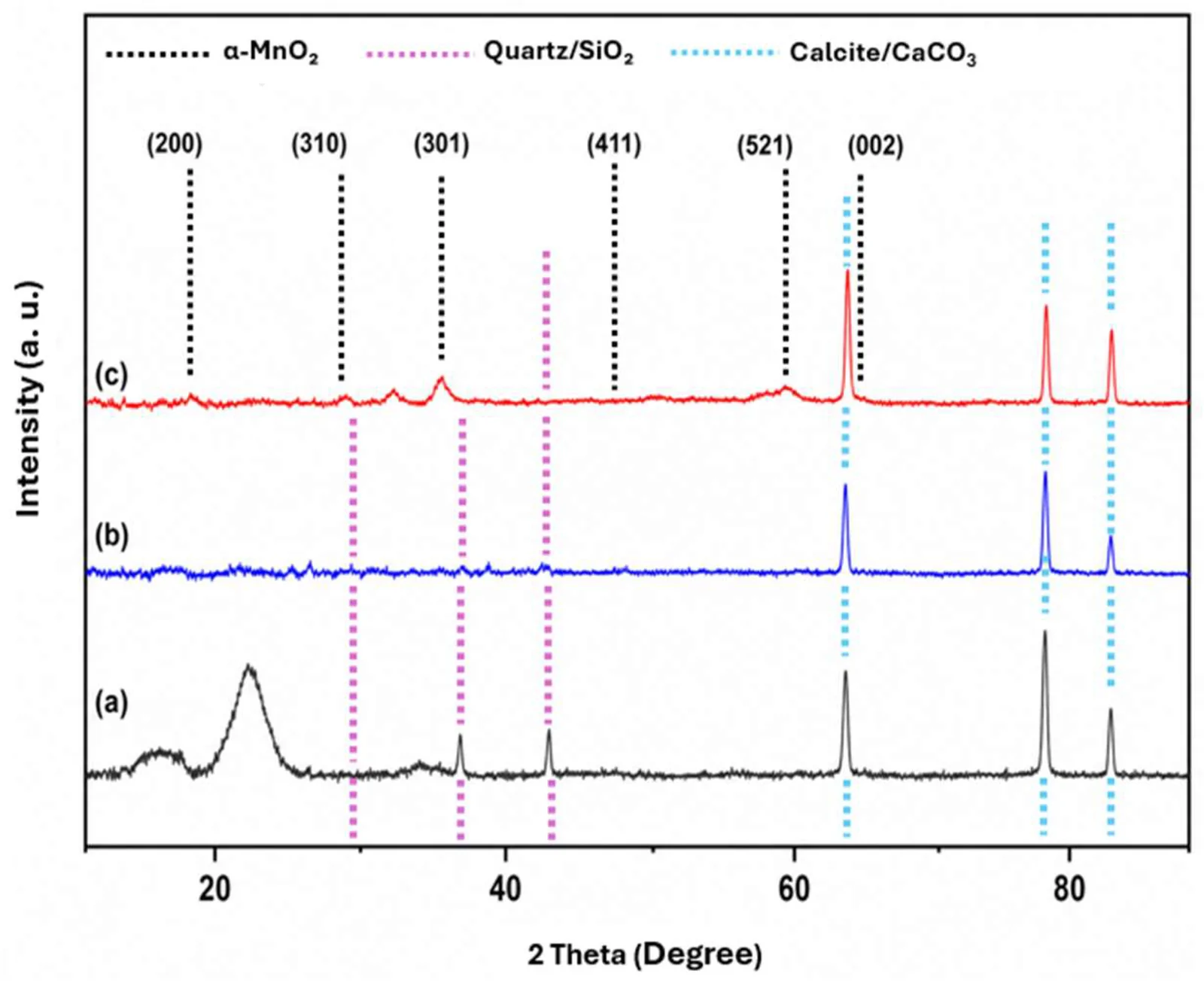
XRD spectra of (**a**) PL, (**b**) biochar, and (**c**) MnO_2_/PL biochar nanocomposite.

The crystalline structure and phase composition of PL, biochar, and the MnO_2_/PL biochar nanocomposite were analyzed by XRD, and the obtained patterns are presented in Figure 3. The diffraction pattern of PL (Figure 3a) exhibited a broad reflection centered around 22° (2θ), which can be attributed to the predominantly amorphous structure of the lignocellulosic biomass [13]. Following pyrolysis, the diffraction pattern of the biochar sample (Figure 3b) showed the disappearance of the broad biomass-related peak, indicating the thermal transformation of the original organic components and the formation of a more carbonized structure. Several diffraction peaks were observed at approximately 29.2°, 37.2°, and 43.3° (2θ), corresponding to quartz (SiO_2_), as assigned in the reference pattern used for Figure 3, while additional reflections at 64.2°, 78.0°, and 83.0° were assigned to calcite (CaCO_3_) phases [19]. The persistence of these mineral phases after carbonization suggests their thermal stability during the pyrolysis process.

The XRD pattern of the MnO_2_/PL biochar nanocomposite (Figure 3c) displayed several additional diffraction peaks located at 18.5°, 29.0°, 37.0°, 50.4°, 60.4°, and 65.1° (2θ), which were indexed to the (200), (310), (301), (411), (521), and (002) crystallographic planes, respectively. These reflections are consistent with the crystalline structure of α-MnO_2_ and show good agreement with the standard JCPDS card No. 44-0141 [20]. The appearance of these additional reflections supports the presence of a crystalline manganese oxide phase in the modified biochar. However, XRD alone cannot unambiguously determine the manganese oxidation state or confirm nanoscale deposition specifically on the biochar surface. Therefore, the identification of α-MnO_2_ is based on comparison of the observed diffraction pattern with the reference data, while the incorporation and spatial distribution of manganese are further supported by the complementary SEM–EDX results. The coexistence of the α-MnO_2_-consistent reflections with quartz and calcite peaks indicates that the biochar support retained after modification with the manganese-containing phase. Further techniques, such as X-ray Photoelectron Spectroscopy (XPS), Raman spectroscopy, and Transmission Electron Microscopy (TEM), would provide additional information on the manganese oxidation state, chemical environment, and nanoscale distribution of the manganese oxide phase.

#### 2.1.4. Surface Area and Porosity Analysis

**Figure 4:**
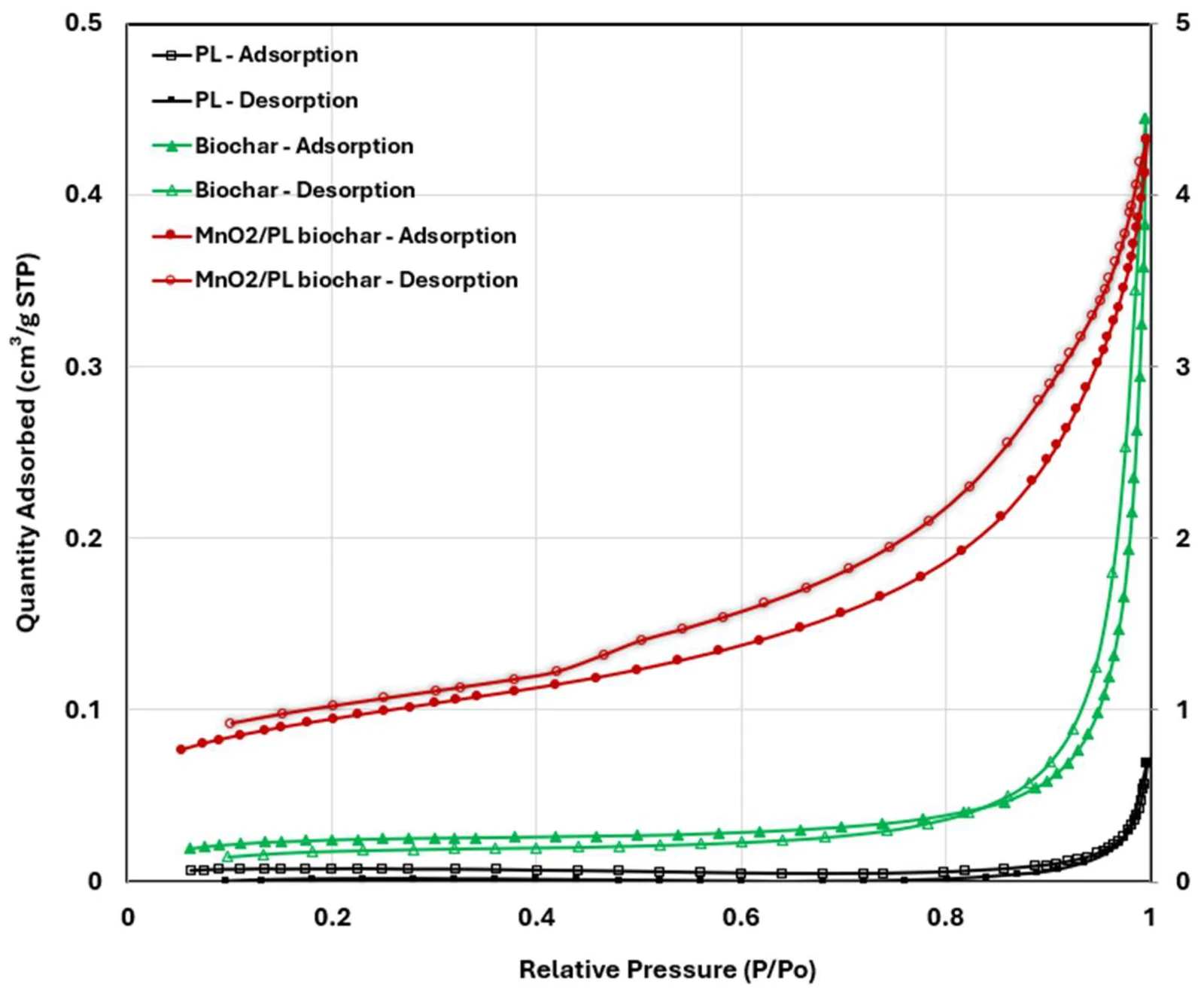
Hysteresis loops of PL, biochar, and MnO_2_/PL biochar.

**Figure 5:**
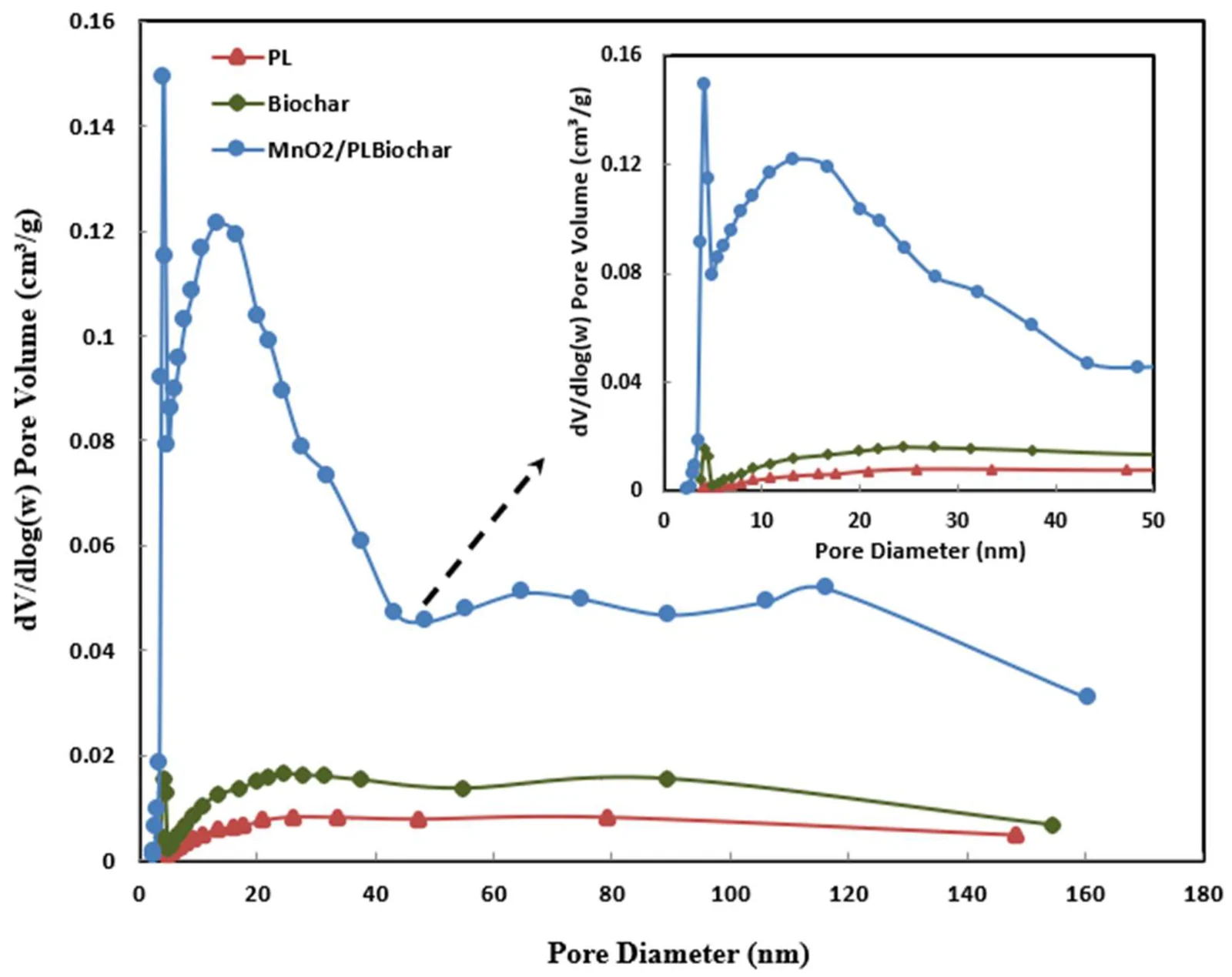
Differential pore size distribution of PL, biochar, and MnO_2_/PL biochar calculated from the nitrogen adsorption data. The inset is a magnified view of the low-pore-diameter region and uses the same ordinate definition and units as the main panel.

Nitrogen adsorption–desorption experiments and pore distribution analysis were employed to characterize the textural features of PL, biochar, and MnO_2_-modified PL biochar, as summarized in Table 1 and Figure 4 and Figure 5. Figure 4 presents the N_2_ adsorption–desorption isotherms of the investigated materials. Both PL and biochar exhibited mixed Type II/IV isotherm characteristics with an H3 hysteresis loop, reflecting the coexistence of irregular macroporous and mesoporous structures within the materials [21]. In comparison, The MnO_2_/PL biochar composite showed typical Type IV isotherm behavior with an H3 hysteresis loop, indicating that MnO_2_ incorporation promoted the generation of a predominantly mesoporous structure [21,22]. The quantitative textural properties are summarized in Table 1. A significant enhancement in surface area and pore volume was observed during the transition from raw biomass to the engineered nanocomposite. Notably, the MnO_2_/PL biochar nanocomposite exhibited a remarkable enhancement in specific surface area, achieving approximately 154-fold and 41-fold increases compared with raw palm leaves and pristine biochar, respectively. Furthermore, the total pore volume exhibited a considerable increase from 0.002 cm^3^/g for the raw PL to 0.012 cm^3^/g for the biochar, reaching a maximum of 0.136 cm^3^/g for the MnO_2_/PL nanocomposite. Across all samples, the average pore diameter remained within the 11–48 nm range, further confirming the mesoporous nature of these adsorbents. This well-developed porosity and high surface area are instrumental in providing the necessary active sites for the efficient sequestration of target contaminants [21].

The pore size distribution curves (Figure 5) further illustrate this transformation. While the raw biomass and biochar exhibited broad and relatively low-intensity distributions, the MnO_2_/PL nanocomposite showed a sharp and intense peak centered at approximately 4 nm, indicating a highly uniform mesoporous structure. The average pore diameter decreased significantly from 48 nm (PL) and 46 nm (biochar) to 11 nm for the nanocomposite. This shift confirms the transition toward a more refined mesoporous structure. The reduction in average pore size suggests that the MnO_2_ nanoparticles are predominantly located within the larger pores of the biochar, effectively partitioning them into smaller, more numerous mesopores [18]. This enhanced surface area and well-developed mesoporous architecture are critical for the efficient diffusion and adsorption of CIP from aqueous solutions.

#### 2.1.5. pH of the Zero Charge Point (*pH_ZC_*)

**Figure 6:**
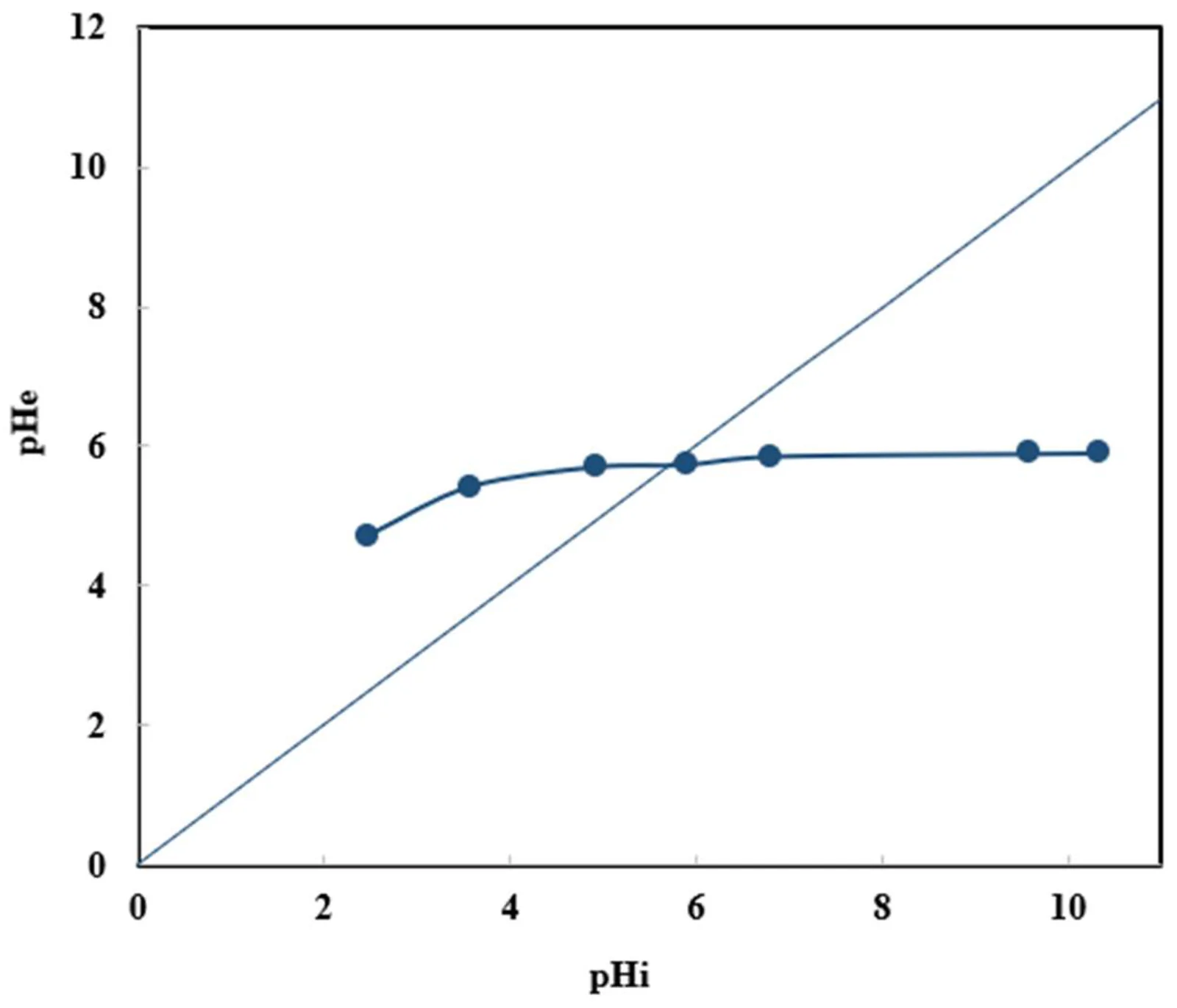
Determination of the point of zero charge ($pH_{pzc}$) of MnO_2_/PL biochar using 0.1 g of adsorbent in 20 mL of 0.1 M NaCl solution.

Figure 6 presents the relationship between the initial and equilibrium pH values obtained after mixing 0.1 g of MnO_2_/PL biochar with 20 mL of 0.1 M NaCl solution. The intersection of the experimental trend with the $pH_{i}=pH_{f}$ reference line was observed at approximately pH 5.8, corresponding to a point of zero charge ($pH_{pzc}$) of about 5.8. Below this pH value, the equilibrium pH increased relative to the initial pH, indicating proton exchange between the solution and the composite surface. Under these conditions, the surface is expected to carry a net positive charge. At initial pH values above approximately 5.8, the equilibrium pH showed only limited variation and remained close to 5.9. Accordingly, the MnO_2_/PL biochar surface is expected to be predominantly positively charged at pH values below $pH_{pzc}$ and negatively charged above it. This behavior is relevant to CIP adsorption because the electrostatic interactions between the adsorbent surface and ionic CIP species depend on the solution pH relative to $pH_{pzc}$.

### 2.2. Adsorption Studies

#### 2.2.1. Effect of pH

**Figure 7:**
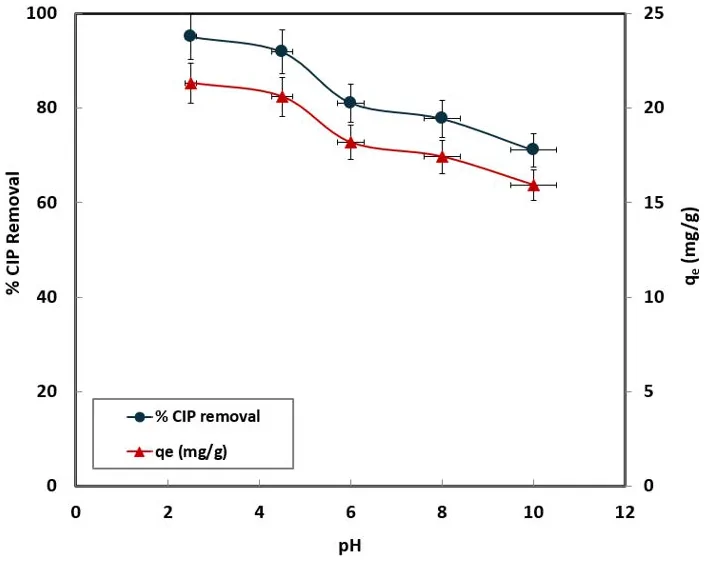
Effect of solution pH on CIP adsorption onto MnO_2_/PL biochar using 0.07 g of adsorbent and 30 mL of CIP solution (52 mg/L) at 298 K, with an agitation speed of 120 rpm and an equilibrium time of 24 h.

The effect of solution pH on CIP adsorption by MnO_2_/PL biochar is shown in Figure 7. CIP removal was highest at pH 2.5 (approximately 95%) and gradually declined to about 71% at pH 10. The equilibrium adsorption capacity (*q_e_*) showed a similar decreasing trend, declining from approximately 21.2 mg/g at pH 2.5 to about 16.0 mg/g at pH 10. This variation reflects the combined influence of CIP speciation and changes in the surface charge of the adsorbent.

CIP is an amphoteric molecule with reported pKa values of approximately 6.1 and 8.7 [23]. It therefore occurs mainly as a cationic species under acidic conditions, predominantly as a zwitterion around neutral pH, and increasingly as an anion under alkaline conditions [23]. The measured ($pH_{pzc}$) of MnO_2_/PL biochar was approximately 5.8 (Figure 6), indicating a predominantly positive surface below this value and a negative surface above it. Thus, at pH 2.5, the positively charged surface and the predominant cationic form of CIP do not favor simple electrostatic attraction, suggesting that the high adsorption observed under acidic conditions arises from several interactions rather than surface charge effects alone.

A similar preference for acidic conditions has been reported for rice-husk-derived activated carbon, for which maximum CIP adsorption occurred at pH 3.0. The authors related this behavior to the interaction between CIP speciation and the charge characteristics of the adsorbent surface, while also noting the influence of competing OH^−^ ions at higher pH [24]. In the present system, the high uptake at pH 2.5 may therefore be associated with hydrogen bonding, π–π interactions between the aromatic structure of CIP and carbon domains, pore diffusion, and interactions of CIP functional groups with Mn–O surface sites. The carbon matrix and manganese oxide phase may act synergistically, with the former providing accessible adsorption domains and the latter contributing additional reactive surface sites.

As the pH increased above the ($pH_{pzc}$), the surface progressively acquired a negative charge, while CIP became increasingly anionic above pH 8.7. Electrostatic repulsion between the adsorbent and anionic CIP may consequently contribute to the lower adsorption observed at alkaline pH. Competition from OH^−^ and changes in the protonation state of surface functional groups may further reduce CIP uptake. These observations indicate that CIP adsorption is controlled by multiple pH-dependent interactions rather than by electrostatic forces alone.

Despite the decrease in adsorption at higher pH, MnO_2_/PL biochar retained appreciable CIP removal across the investigated range, demonstrating its potential for application under different aqueous conditions.

#### 2.2.2. Effect of CIP Initial Concentration

**Figure 8:**
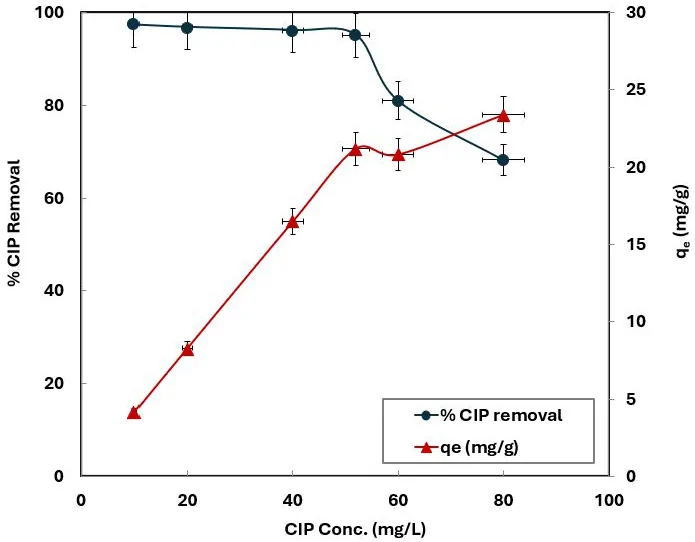
Effect of initial CIP concentration on CIP uptake by MnO_2_/PL biochar at pH 2.5, using 0.07 g of adsorbent and 30 mL of CIP solution at 298 K, with an agitation speed of 120 rpm and an equilibrium time of 24 h.

Figure 8 illustrates the impact of varying initial CIP concentrations on the adsorption efficiency of the MnO_2_/PL biochar nanocomposite. The composite maintained a high removal efficiency of above 95% across the concentration range of 10–50 mg/L, indicating a strong affinity toward CIP molecules. Over the same concentration range, the adsorption capacity increased progressively from approximately 4 mg/g at 10 mg/L to about 21 mg/g at 50 mg/L. This increase can be attributed to the greater concentration gradient, which provides an increased driving force for mass transfer of CIP molecules from the solution to the available adsorption sites. At higher initial concentrations, the adsorption capacity continued to increase slightly, reaching approximately 23 mg/g at 80 mg/L, whereas the removal efficiency decreased to nearly 81% and 68% at 60 and 80 mg/L, respectively. This contrasting behavior indicates that, although a greater amount of CIP was adsorbed per unit mass of adsorbent at higher concentrations, the available adsorption sites became progressively saturated and were insufficient to remove the same proportion of CIP from solution.

The porous carbon matrix, together with oxygen-containing functional groups and manganese oxide-related surface sites, provides multiple potential sites for CIP interactions [14,25]. However, as the initial CIP concentration increased, competition among CIP molecules for the available adsorption sites became more pronounced. Consequently, a larger fraction of the pollutant remained in solution, resulting in reduced removal efficiency. The gradual increase and eventual tendency toward stabilization of $q_{e}$ further suggest progressive occupation of the available adsorption sites as the CIP concentration increased. Similar concentration-dependent behavior has been reported for CIP and other antibiotics using biochar-based adsorbents [26]. This finding demonstrates that the MnO_2_/PL biochar exhibits high CIP removal at moderate concentrations while maintaining an increasing adsorption capacity under higher pollutant loading conditions.

#### 2.2.3. Effect of MnO_2_/PL Biochar Dose

**Figure 9:**
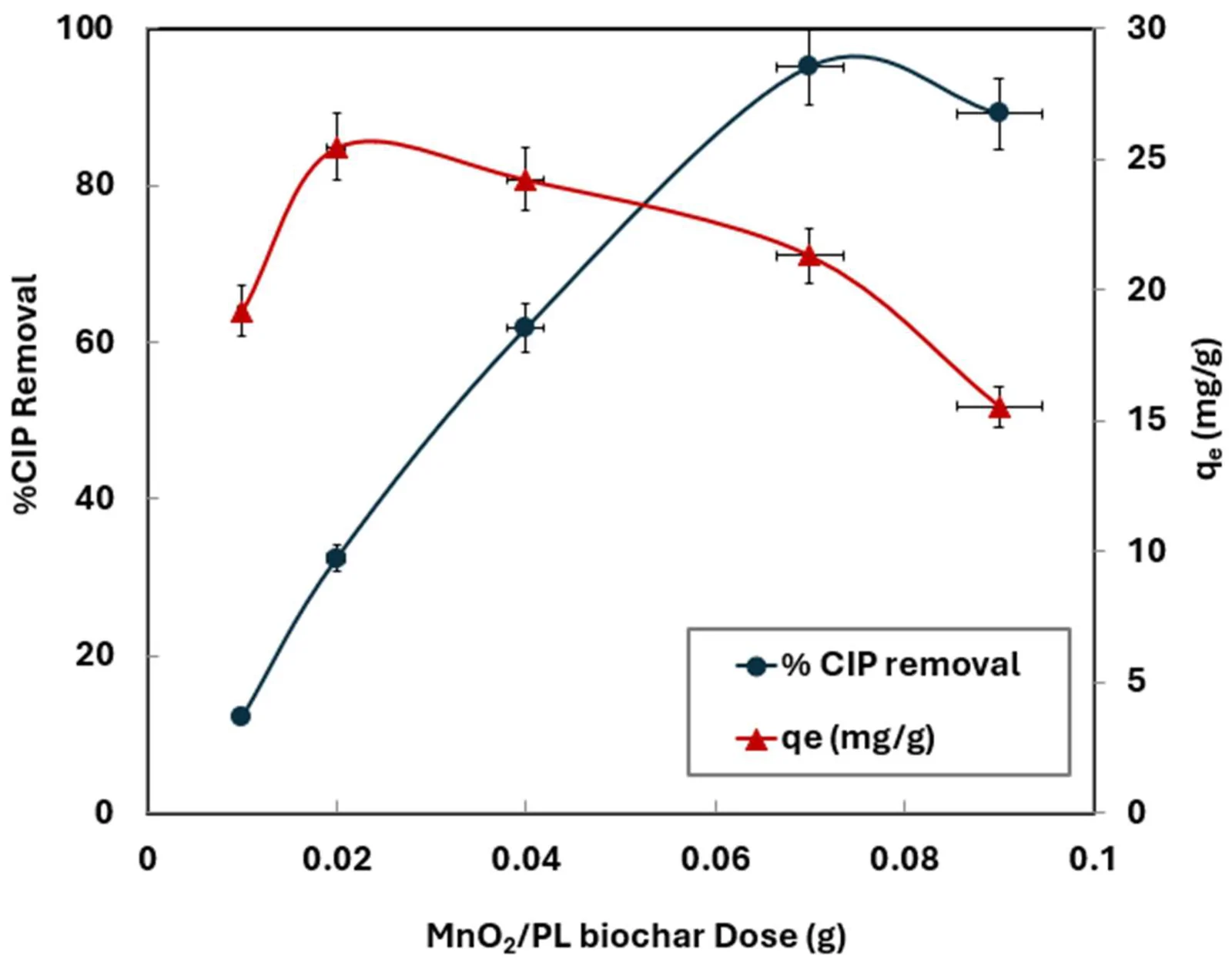
Effects of different MnO_2_/PL biochar doses on CIP uptake at pH 2.5, using 30 mL of CIP solution (52 mg/L) at 298 K, with an agitation speed of 120 rpm and an equilibrium time of 24 h.

The effect of MnO_2_/PL biochar dosage on CIP removal is shown in Figure 9. A gradual increase in adsorption efficiency was observed as the adsorbent mass increased from 0.01 to 0.07 g, where CIP removal improved from nearly 12% to 95%. The *q_e_* increased from approximately 20.0 mg/g at 0.01 g to a maximum of about 25.5 mg/g at 0.02 g. The initial increase in CIP removal can be attributed to the greater availability of accessible active sites and reactive surface functionalities as the adsorbent dosage increased. Moreover, the increased availability of porous structures provides additional contact regions for CIP molecules, enabling interactions with the composite surface through hydrogen bonding, π–π stacking, and coordination/surface complexation processes involving MnO_2_ nanoparticles and carbon-based domains. Similar improvements in antibiotic removal with increasing biochar dosage have been reported for modified biochar adsorbents [5]. A minor reduction in CIP removal efficiency was noted when the MnO_2_/PL biochar dosage increased beyond 0.07 g, with the removal percentage decreasing from approximately 95% to 89% at 0.09 g. This decrease was also reflected in *q_e_*, which declined from approximately 21.5 mg/g at 0.07 g to 15.5 mg/g at 0.09 g. Although increasing the adsorbent dosage provides a greater number of potential adsorption sites, the fixed amount of CIP in solution becomes distributed over a larger adsorbent mass, resulting in lower adsorption capacity on a unit-mass basis. At the highest dosage, particle aggregation may also reduce the effective surface area and accessibility of some adsorption sites, thereby limiting their utilization. Furthermore, excessive adsorbent loading may promote interactions among biochar particles and partial overlap or shielding of active sites, reducing the effective contact between CIP molecules and the adsorbent surface. Similar observations have been reported for antibiotic adsorption using biochar-based materials, where an optimum adsorbent dosage is achieved before reduced site utilization and possible particle aggregation begin to affect removal efficiency [26]. This result demonstrates that optimizing the adsorbent dosage is essential for maximizing adsorption performance while avoiding unnecessary material consumption and minimizing inefficient utilization of adsorption sites.

#### 2.2.4. Adsorption Kinetics

**Figure 10:**
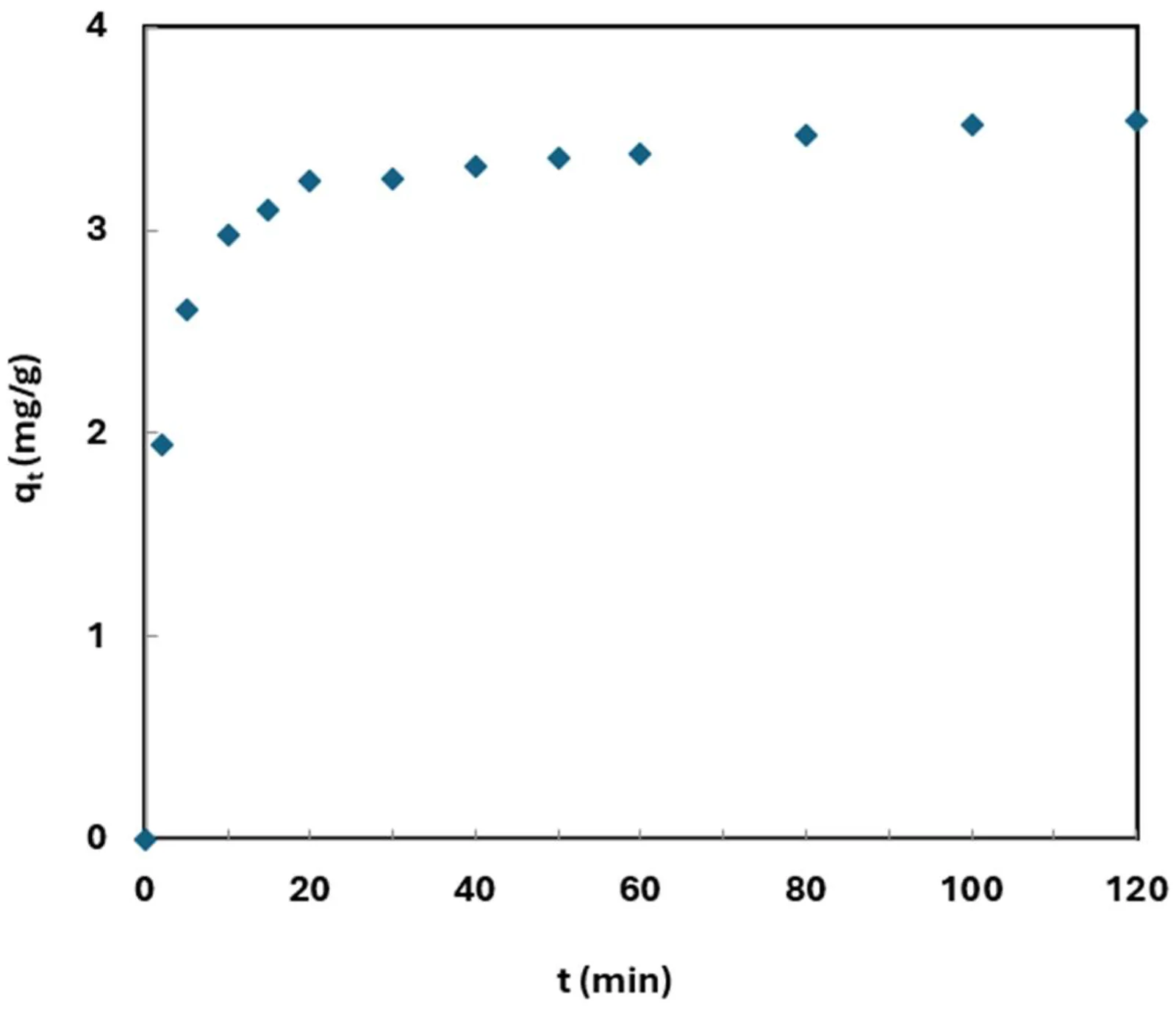
Effect of contact time on the adsorption capacity ($q_{t}$) of CIP by MnO_2_/PL biochar at pH 2.5, using 0.07 g of adsorbent and 30 mL of CIP solution (52 mg/L) at 298 K and an agitation speed of 120 rpm.

Adsorption kinetic analysis is important for elucidating the adsorption rate, identifying the controlling mechanisms, and evaluating the interactions occurring between CIP molecules and the MnO_2_/PL biochar nanocomposite. The relationship between adsorption capacity ($q_{t}$) and contact time is shown in Figure 10. CIP uptake was relatively fast at the beginning, with $q_{t}$ increasing from about 1.9 mg/g at 5 min to 3.0 mg/g at 15 min. The increase subsequently became less pronounced, reaching approximately 3.3 mg/g at 40–60 min and 3.5 mg/g at 100–120 min. This trend indicates that the most accessible adsorption sites were occupied rapidly, followed by a slower uptake stage as site availability decreased.

After approximately 60 min, only a minor increase in $q_{t}$ was observed, suggesting that the system was approaching equilibrium. The initial rapid uptake may be related to CIP transport to readily available surface sites, whereas the later stage may involve slower diffusion into less accessible pores and interaction with remaining adsorption sites.

**Figure 11:**
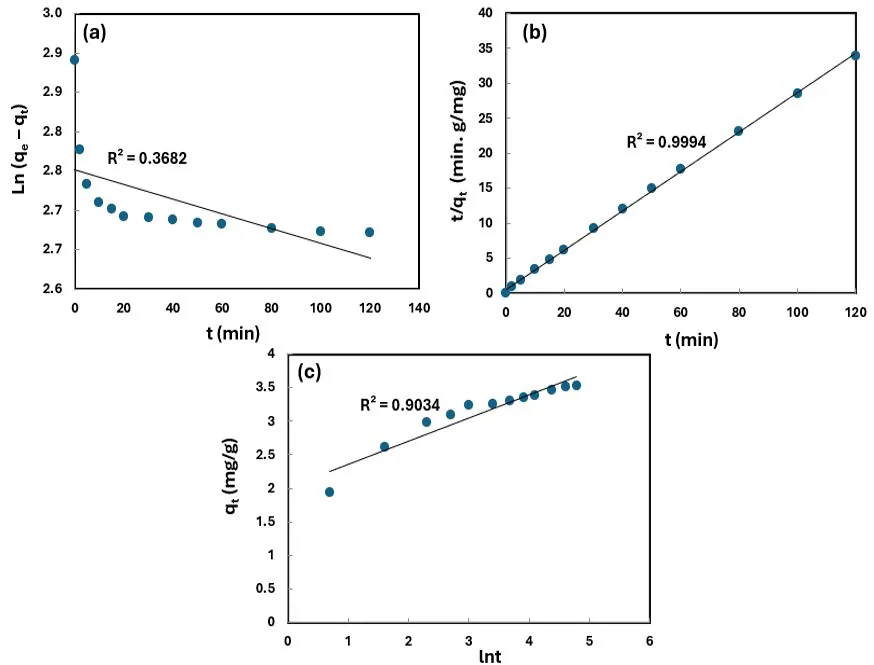
Kinetic model fitting curves for CIP adsorption onto MnO_2_/PL biochar based on (**a**) PFO, (**b**) PSO, and (**c**) Elovich equations.

The kinetic data were further evaluated using the pseudo-first-order (PFO), pseudo-second-order (PSO), and Elovich kinetic models, as described by Equations (1)–(3) [23,27]. The regression plots for the applied models are illustrated in Figure 11, whereas the estimated kinetic constants and correlation coefficients are listed in Table 2.

The PFO model:

(1) $$ln(q_{e}-q_{t})=lnq_{e}-k_{1}t$$

where $q_{e}$ and $q_{t}$ (mg/g) represent the adsorption capacities of CIP at equilibrium and at a given adsorption time t, respectively, while $k_{1}$ (min^−1^) denotes the rate constant associated with the PFO.

The PSO model:

(2) $$\frac{t}{q_{t}}=\frac{1}{k_{2}q_{e}^{2}}+\frac{t}{q_{e}}$$

where $k_{2}$ (g/mg min) is the PSO rate constant.

The Elovich model:

(3) $$q_{t}=\frac{1}{\beta }ln(\alpha \beta )+\frac{1}{\beta }lnt$$

where α (mg/g min) is the initial adsorption rate, and β (g/mg) is the Elovich constant related to surface coverage and activation energy for chemisorption.

As shown in Table 2, the experimental kinetic data were most accurately represented by the PSO model, which exhibited the highest correlation coefficient (*R*^2^ = 0.999) compared with the PFO and Elovich models (*R*^2^ = 0.368 and 0.903, respectively). The excellent fitting of the PSO model indicates its greater suitability for predicting CIP adsorption behavior. This result implies that the adsorption rate is closely related to the availability of reactive surface sites and the interactions occurring between CIP molecules and functional groups present on the MnO_2_/PL biochar composite. However, a good fit to the PSO model does not necessarily indicate that chemisorption is the only controlling mechanism, since this model can also describe adsorption systems involving multiple interaction pathways. The adsorption of CIP onto MnO_2_/PL biochar is therefore likely governed by a combination of interactions, including hydrogen bonding, π–π electron donor–acceptor interactions between CIP aromatic structures and the carbon matrix, and surface complexation involving CIP functional groups and Mn–O active sites [2,27].

The calculated PSO rate constant (*k*_2_) for the MnO_2_/PL biochar was 0.138 g/mg·min, indicating a relatively rapid adsorption process during the initial stages of contact. While the Elovich model provided a lower *R*^2^ value compared to the PSO model, its relatively high correlation (0.903) suggests that the adsorbent surface is energetically heterogeneous. The Elovich parameters, α = 111.7 mg/g·min and β = 2.88 g/mg, further support the idea that the MnO_2_/PL biochar possesses a variety of active sites with different binding energies, likely due to the combination of the carbonaceous biochar framework and the greenly synthesized MnO_2_ nanoparticles [27].

#### 2.2.5. Adsorption Isotherms

**Figure 12:**
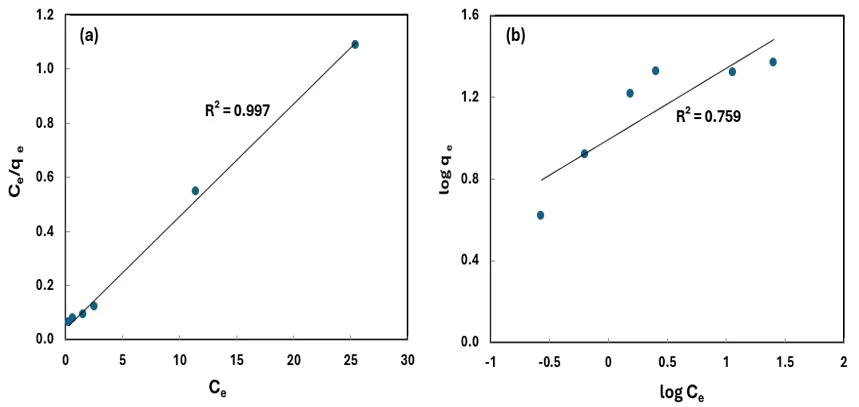
Linearized adsorption isotherm plots for CIP uptake by MnO_2_/PL biochar nanocomposite: (**a**) Langmuir model and (**b**) Freundlich model.

CIP adsorption equilibrium on the MnO_2_/PL biochar nanocomposite was analyzed by applying the Langmuir and Freundlich adsorption isotherms (Equations (4) and (5)) [4,23]. The corresponding linear regression plots are illustrated in Figure 12, and the estimated isotherm constants and fitting parameters are provided in Table 3.

The linearized equation of the Langmuir isotherm model is given as follows

(4) $$\frac{C_{e}}{q_{e}}=\frac{1}{Q_{max}b}+\frac{C_{e}}{Q_{max}}$$

where $Q_{max}$ (mg/g) is the maximum monolayer adsorption capacity, *b* (L/mg) is the Langmuir constant associated with adsorption affinity. The Freundlich model is stated as follows:

(5) $$\log q_{e}=\log K_{F}+\frac{1}{n}\log C_{e}$$

where *K_F_* is the Freundlich constant associated with adsorption capacity and 1/*n* stands for surface heterogeneity and adsorption intensity.

The isotherm fitting results demonstrated the good applicability of the Langmuir model, which achieved a higher correlation coefficient (*R*^2^ = 0.997) compared with the Freundlich model (*R*^2^ = 0.759). To provide a more comprehensive assessment of model suitability, the root mean square error (RMSE) and chi-square ($\chi^{2}$) values were also calculated. The Langmuir model showed lower RMSE and $\chi^{2}$ values (1.963 and 1.520, respectively) than the Freundlich model (4.845 and 8.829, respectively), confirming its better agreement with the experimental equilibrium data. This strong Langmuir correlation implies that CIP uptake by the MnO_2_/PL biochar nanocomposite is governed by monolayer adsorption on relatively uniform and equivalent surface sites. The results suggest that adsorption occurs through the occupation of discrete active sites, where each site accommodates one CIP molecule without interaction between adjacent adsorbed molecules [4].

The $Q_{max}$ was calculated as 24 mg/g at 25 °C. The Langmuir affinity constant (b=0.983 L/mg) reflects the strong interaction between CIP molecules and the active sites of the MnO_2_/PL biochar. Furthermore, the Freundlich parameter (1/n=0.348) being below unity confirms the favorable nature of the adsorption process and indicates the high affinity of the composite toward CIP molecules, particularly at relatively low pollutant concentrations [4,28].

The isotherm results are highly consistent with the PSO kinetic model discussed previously. Both the Langmuir fit and the PSO model suggest that the removal of CIP is dominated by chemisorption and specific surface interactions. The high surface area and well-developed mesoporosity (as confirmed by BET analysis) facilitate the rapid transport and subsequent monolayer binding of CIP via electrostatic attraction, π−π stacking, and hydrogen bonding [28].

#### 2.2.6. Comparison with Other Adsorbents

The adsorption capacity of the MnO_2_/PL biochar nanocomposite was further assessed by comparing its maximum Langmuir adsorption capacity ($Q_{\max}$) with those of different adsorbent materials previously reported for CIP removal. As summarized in Table 4, the MnO_2_/PL biochar exhibited a $Q_{\max}$ value of 24 mg/g, which is comparable to many biochar-derived and other economical adsorbents. This result highlights the effectiveness of the synthesized nanocomposite and its potential application as a sustainable adsorbent for the treatment of pharmaceutical-contaminated wastewater.

#### 2.2.7. Adsorption Mechanism of CIP onto MnO_2_/PL Biochar

The adsorption mechanism of CIP on the MnO_2_/PL biochar nanocomposite involves the combined effects of several interactions between the antibiotic molecules and the functionalized composite surface. The BET results revealed a substantial enhancement in specific surface area following MnO_2_ loading, suggesting the formation of a more developed porous network with improved accessibility and an increased number of active adsorption sites. This enhanced porosity facilitates the diffusion and retention of CIP molecules through pore filling and physical interactions. Furthermore, the oxygen-containing functional groups identified by FTIR provide active sites for hydrogen bonding with the carboxyl, carbonyl, and piperazine groups of CIP [33]. The aromatic domains within the biochar matrix can also favor π–π electron donor–acceptor interactions with the quinolone ring of CIP, thereby promoting its association with the carbonaceous surface [1].

The XRD results showed crystalline MnO_2_ phases in the composite, while SEM–EDX further confirmed the incorporation of Mn-containing phases. These manganese oxide domains may provide additional reactive sites for interaction with CIP through possible surface complexation involving Mn–O/Mn–OH groups and the oxygen- and nitrogen-containing functional groups of CIP [26]. The experimentally determined $pH_{pzc}$ of approximately 5.8 provides additional information on the pH-dependent surface charge of the composite. Below this value, the surface is expected to be predominantly positively charged, whereas it becomes increasingly negatively charged above $pH_{pzc}$. However, because zeta-potential measurements were not performed, the magnitude of the surface charge and its direct contribution to CIP adsorption cannot be established. Therefore, electrostatic attraction or repulsion is considered a possible contributing factor rather than a confirmed adsorption mechanism.

Overall, the observed CIP removal is likely associated with the combined effects of the developed porous structure and multiple surface interactions, including hydrogen bonding, π–π interactions, pore diffusion, and possible complexation with MnO_2_-derived surface sites. The contribution of electrostatic interactions may vary with solution pH and CIP speciation, but further surface-sensitive characterization would be required to determine the relative importance of each mechanism.

#### 2.2.8. Adsorption–Desorption and Reusability

**Figure 13:**
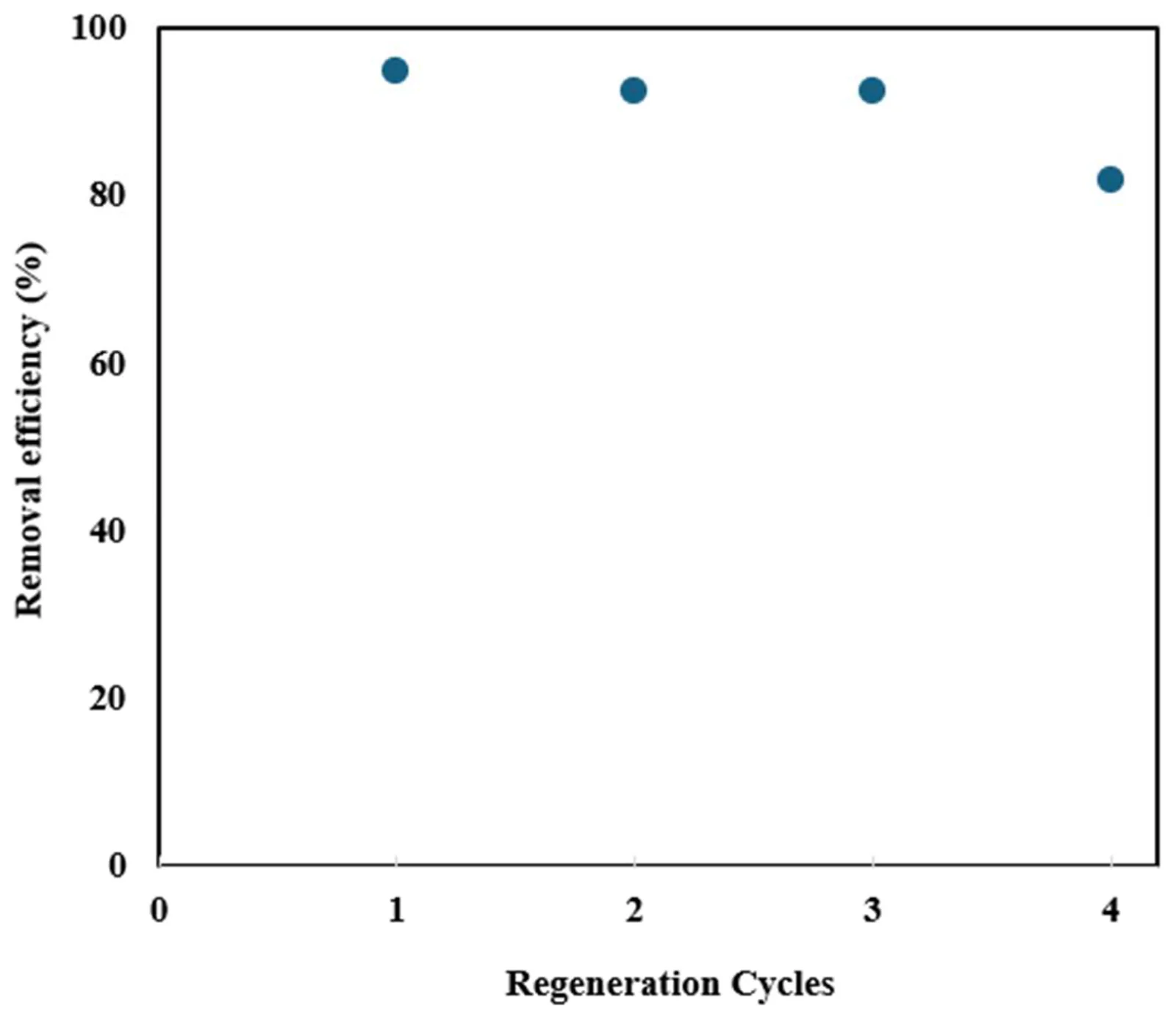
Recycling of MnO_2_/PL biochar nanocomposite for multiple adsorption–desorption cycles of CIP using 0.07 g of adsorbent at pH 2.5, with 30 mL of CIP solution (52 mg/L), at 298 K, an agitation speed of 120 rpm, and an equilibrium time of 24 h.

The regeneration and repeated-use performance of the MnO_2_/PL biochar nanocomposite was investigated over four successive adsorption–desorption cycles, with the results presented in Figure 13. The composite achieved approximately 95% CIP removal in the first cycle. Following regeneration, the removal efficiency remained relatively stable, reaching about 92–93% in the second and third cycles, before decreasing to about 81% in the fourth cycle. Overall, the removal efficiency decreased by approximately 14 percentage points after four cycles, indicating that a substantial portion of the adsorption capacity was retained during repeated use. The gradual loss in efficiency may be related to incomplete release of CIP during the regeneration step, resulting in residual molecules occupying part of the pore network or active adsorption sites. Other possible factors include the removal of loosely bound material during washing, minor changes in surface functionalities, or reduced accessibility of some pores after repeated treatment. Such effects can influence the regeneration behavior of biochar-based and metal-oxide-modified adsorbents because their performance depends on both the porous carbon structure and the reactive sites associated with the incorporated metal oxide [21].

Despite this decrease, the MnO_2_/PL biochar maintained a high proportion of its initial CIP removal efficiency over the four cycles, indicating promising short-term reusability. The observed retention is consistent with trends reported for modified and magnetic biochar-based adsorbents that preserve substantial adsorption performance following repeated regeneration [21].

The potential for manganese release was evaluated thermodynamically under the optimum adsorption condition of pH 2.5. Although MnO_2_ is a sparingly soluble solid, its acidic dissolution involves the reduction of Mn(IV) to Mn(II), coupled with proton transfer and dissolution of the oxide [34]. Therefore, a redox-equilibrium approach was used to estimate the equilibrium concentration of dissolved Mn^2+^ under the specified pH and redox conditions. This calculation is not intended to represent a conventional Ksp-based solubility limit; rather, it provides a thermodynamic screening assessment of potential Mn release under the specified oxygen-equilibrated conditions and is not a direct measurement of Mn leaching from the MnO_2_/PL biochar composite.

For the oxygen reduction reaction,

O_2_(g) + 4H^+^(aq) + 4e^−^ ⇌ 2H_2_O (l)

The equilibrium potential at 298 K, assuming an oxygen partial pressure of $p_{O_{2}}=1$ atm and pH 2.5, is approximately 1.08 V. This value represents the equilibrium redox potential of the oxygen/water couple under the specified acidic and oxygen-equilibrated conditions.

For MnO_2_ reduction,

MnO_2_ (s) + 4H^+^ (aq) + 2e^−^ ⇌ Mn^2+^ (aq) + 2H_2_O (l)

The Nernst equation at 298 K can be written as [35]:

(6) $$E=E^{o}-\frac{0.05916}{2}log(\frac{\left[{Mn}^{2+}\right]}{{\left[H^{+}\right]}^{4}})$$

where (*E*) is the equilibrium electrode potential (V), (*E*^o^) is the standard reduction potential of the MnO_2_/Mn^2+^ couple (V), (Mn^2+^) is the molar concentration of dissolved Mn^2+^ (mol/L), and (H^+^) is the hydrogen-ion concentration (mol/L).

Taking the standard reduction potential of the MnO_2_/Mn^2+^ couple as (*E*^o^ = 1.23) V [36], the oxygen/water equilibrium potential as (*E* = 1.08) V at pH 2.5, and the hydrogen-ion concentration calculated from the experimental pH as follows:

Since the experimental pH is 2.5,

(7) $$[H^{+}]=10^{-pH}$$

$$[H^{+}]=3.16\times 10^{-3}\;\mathrm{mol}/L$$

Substitution of these values into Equation (6) gives:

$$1.08=1.23-\frac{0.05916}{2}log(\frac{{[Mn}^{2+}]}{{\left[3.16\times 10^{-3}\right]}^{4}})$$

$$log(\frac{\left[{\mathrm{Mn}}^{2+}\right]}{{\left[3.16\times 10^{-3}\right]}^{4}})=5.071$$

$$[{\mathrm{Mn}}^{2+}]=1.18\times 10^{-5}\;\mathrm{mol}/L$$

Using the molar mass of Mn (54.94 g/mol), the corresponding theoretical equilibrium concentration of dissolved Mn^2+^ is approximately 0.65 mg/L Mn under the specified acidic and oxygen-equilibrated conditions.

The calculated concentration represents an idealized thermodynamic equilibrium estimate under the specified acidic and oxygen-equilibrated conditions. The calculation assumes ideal solution behavior, with dissolved-species activities approximated by their molar concentrations and unit activity for solid MnO_2_ and liquid water. These assumptions are appropriate for a thermodynamic screening assessment but do not reproduce the complete chemical environment of the adsorption system.

The relevance of evaluating MnO_2_ stability under the strongly acidic optimum adsorption condition (pH 2.5) is supported by previous research. Aguiar et al. [37] investigated MnO_2_-based treatment of acidic mine water with an initial pH of approximately 2.7. Their results demonstrated the importance of pH in Mn-containing MnO_2_ systems, with effective manganese removal obtained following adjustment toward near-neutral conditions. The study also reported changes in the Mn-containing solid phase following treatment, with hausmannite (Mn_3_O_4_) detected after interaction with the Mn-containing solution [37]. Although their system differs from the present MnO_2_/PL biochar composite and did not directly quantify Mn leaching from the adsorbent, these findings support consideration of pH-dependent MnO_2_ stability and possible solid-phase transformations in Mn-based water treatment materials.

For environmental relevance, the calculated value was compared with both international guideline values and the applicable Saudi wastewater discharge requirement. The World Health Organization (WHO) guideline value for manganese in drinking water is 0.08 mg/L, while the U.S. Environmental Protection Agency (US EPA) secondary maximum contaminant level is 0.05 mg/L. In addition, because the present material is intended for wastewater treatment, the applicable Saudi wastewater discharge requirement was considered; the specified limit for Mn in treated wastewater is 0.2 mg/L (annual average of monthly samples) [38]. The theoretical equilibrium concentration of 0.65 mg/L is therefore approximately 8.1, 13.0, and 3.25 times the WHO, US EPA, and Saudi regulatory values, respectively. These comparisons do not indicate that 0.65 mg/L Mn would actually be released from the composite, because the calculated value represents an idealized thermodynamic equilibrium estimate rather than an experimental leaching concentration. Nevertheless, the result indicates that the potential for Mn release under the acidic optimum adsorption condition warrants experimental verification and cannot be considered negligible solely on the basis of MnO_2_ incorporation into the adsorbent.

Actual Mn release from the MnO_2_/PL biochar composite may be influenced by the MnO_2_ phase and crystallinity, its interaction with the biochar matrix, solution ionic strength, complexation reactions, competing ions, dissolved organic matter, and the actual redox conditions during adsorption. In addition, thermodynamic calculations do not account for kinetic limitations that may restrict MnO_2_ dissolution.

Accordingly, the present calculation provides a transparent assessment of the potential for secondary Mn contamination while recognizing the limitation that direct Mn-leaching measurements were not performed. Quantitative determination of dissolved Mn by Inductively Coupled Plasma–Optical Emission Spectrometry (ICP-OES) or Atomic Absorption Spectrometry (AAS) is therefore required to establish the actual Mn release under the experimental adsorption conditions. Further investigations should evaluate Mn release during repeated regeneration cycles, characterize the regenerated adsorbent, and assess treatment performance and Mn stability using real wastewater matrices. Such studies would provide stronger evidence regarding the long-term stability and environmental performance of the MnO_2_/PL biochar composite.

## 3. Materials and Methods

### 3.1. Materials

Palm leaves (PL), serving as the biomass precursor, were sourced from a local farm in Al-Ahsa, Saudi Arabia. Manganese chloride tetrahydrate (MnCl_2_·4H_2_O, 98%) was utilized as the manganese precursor for synthesizing the MnO_2_/PL biochar nanocomposite. The pH of the solutions was modified utilizing 0.1 M HCl and 0.1 M NaOH solutions to obtain the specified values. Ciprofloxacin (CIP, with 98% purity) was chosen as the representative antibiotic for the adsorption investigations. All chemicals used in this study, were obtained from Merck, Darmstadt, Germany.

### 3.2. Preparation of Acacia Nilotica Extract

Pods of *Acacia nilotica* were obtained from local suppliers in Hofuf, Saudi Arabia, and subjected to sequential washing with tap water and distilled water to eliminate attached debris and unwanted materials prior to extraction. A total of 10 g of the cleaned pods was combined with distilled water and subsequently heated at 100 °C for 15 min. The resulting aqueous extract was then subjected to centrifugation at 10,000 rpm for 10 min using an SVGXC-L6Z centrifuge (SVG, Shanghai, China). Following centrifugation, the supernatant was collected and further purified by filtration before its subsequent use.

### 3.3. Synthesis of PL Biochar

The PL biomass underwent rigorous cleaning with distilled water on multiple occasions to eliminate surface contaminants and impurities. Following a 24-h air-drying period, the material was mechanically pulverized, and particles within the 125 to 250 µm size range were isolated for subsequent processing. Approximately 60 g of this prepared PL powder was transformed into biochar via pyrolysis in a tubular furnace operating at 600 °C for 60 min, sustained under a continuous nitrogen atmosphere (200 mL/min). A constant heating rate of 10 °C min^−1^ was applied during the carbonization procedure, and the obtained biochar exhibited a final yield of 38.5%.

### 3.4. Synthesis of MnO_2_/PF Biochar Nanocomposite

**Figure 14:**
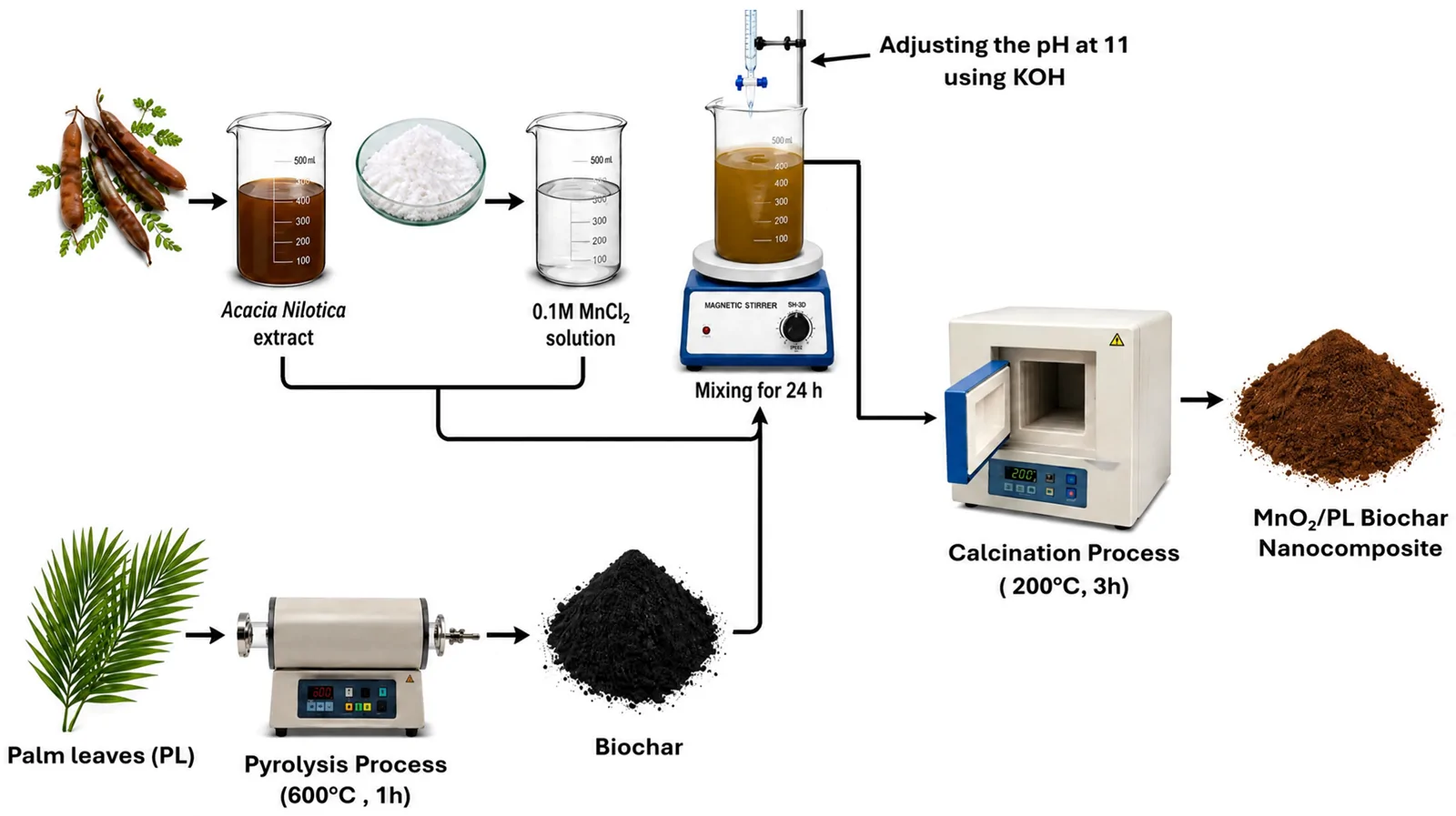
Synthesis of MnO_2_/PL biochar nanocomposite.

The MnO_2_/PL biochar nanocomposite was prepared following our previously established synthesis procedure [39], with the main steps illustrated in Figure 14. Initially, 2.5 g of the pre-processed PL biochar were combined with 100 mL of a 0.1 M MnCl_2_·4H_2_O solution. Afterward, 20 mL of *Acacia nilotica* extract was introduced into this mixture, which was then subjected to continuous stirring for 30 min. The pH of this resulting blend was meticulously adjusted to 11 using a 1 M KOH solution, with monitoring provided by a pH meter. Following this pH adjustment, the mixture continued to stir at room temperature for a duration of 24 h, maintained at a constant speed of 70 rpm. Upon finishing the mixing phase, the resultant precipitate was gathered using filtration, thereafter, undergoing multiple washings with distilled water and ethanol to eliminate remaining contaminants. The refined substance was desiccated at 40 °C and subjected to calcination at 200 °C for 3 h, yielding the final MnO_2_/PL biochar nanocomposite.

### 3.5. Instrument Analysis

The morphological characteristics of the PL and the synthesized MnO_2_/PL biochar nanocomposite were examined via scanning electron microscopy (SEM, FEI QUANTA FEG 250, FEI Company, Hillsboro, OR, USA), which was equipped with an energy-dispersive X-ray spectroscopy (EDX) system for elemental analysis. Porosity characteristics and specific surface areas were determined through the Brunauer–Emmett–Teller (BET, ASAP 2020, Micromeritics Instrument Corporation, Norcross, GA, USA). FTIR spectroscopy (Cary 630, Agilent Technologies, Santa Clara, CA, USA) was employed to identify the surface functional groups present in the prepared materials, while XRD analysis was performed using an XRD-7000 diffractometer (Shimadzu, Tokyo, Japan) with a Cu detector and Cu Kα radiation (λ = 1.54 Å) to determine the crystal structure and phase composition of the MnO_2_/PL biochar nanocomposite.

### 3.6. Batch Adsorption Experiments

The effectiveness of the produced MnO_2_/PL biochar in eliminating CIP from aqueous solutions was evaluated using batch adsorption tests. 50 mL Erlenmeyer flasks with a working capacity of 30 mL CIP solution were used for batch adsorption studies. A magnetic stirrer was used to ensure steady agitation at 100 rpm. Baseline adsorption conditions were established at 298 K and 24 h equilibrium time using 0.07 g of adsorbent and 30 mL of CIP solution (52 mg/L) adjusted to pH 2.5. The effect of pH variation on adsorption behavior was evaluated over the range of 2.5–10 by regulating the solution pH with 0.1 M HCl and NaOH.

The influence of adsorbent dosage was studied by adding varying amounts of nanocomposite (between 0.01 and 0.09 g). For the initial concentration studies, the CIP levels were varied from 10 to 80 mg/L. During the kinetic experiments, aqueous samples were collected at specified intervals (0, 2, 5, 10, 15, 20, 30, 40, 50, 60, 80, 100, and 120 min) and subsequently filtered before analysis. The concentration of unadsorbed CIP in the filtrate was determined based on its maximum absorbance peak at 464 nm using a Shimadzu UV–Vis spectrophotometer (Kyoto, Japan). The regeneration behavior of the MnO_2_/PL biochar was examined through four successive adsorption–desorption cycles. Following each adsorption step, the dye-containing material was treated with 0.1 M NaOH to release the adsorbed dye. The regenerated adsorbent was subsequently rinsed with distilled water and then treated with 0.1 M HCl until the washing solution reached approximately neutral pH (~7.0). The recovered material was reused in the following cycle to assess its regeneration and reusability.

Every adsorption experiment was carried out twice, and data analysis was performed using the average values. The following formulas were used to calculate the equilibrium adsorption capacity (*q_e_*, mg/g) and CIP removal efficiency:

(8) $$\%Removal\;of\;CIP=\frac{\left(C_{0}-C_{t}\right)}{C_{0}}\times 100$$

(9) $$Adsorption\;capacity\left(q_{e}\right)=\frac{\left(C_{0}-C_{e}\right)V}{W}$$

In these equations, *C*_0_ and *C_e_* (mg/L) refer to the initial and equilibrium CIP concentrations, respectively, whereas *C_t_* (mg/L) indicates the CIP concentration measured at a given adsorption time (*t*). *V* represents the solution volume (L), and *W* denotes the amount of MnO_2_/PL biochar nanocomposite used (g).

## 4. Conclusions

A sustainable MnO_2_/PL biochar nanocomposite was successfully prepared using an environmentally friendly synthesis route, where *A. nilotica* pod extract served as a natural reducing and stabilizing agent. The successful generation and anchoring of MnO_2_ nanoparticles onto the palm leaf biochar matrix were verified through FTIR and XRD characterization, confirming the formation of the desired composite structure, while SEM observations revealed a porous structure with well-dispersed nanoparticles that provided abundant adsorption sites. Batch adsorption experiments demonstrated excellent adsorption performance toward CIP, achieving a maximum removal efficiency of approximately 95%. The experimental adsorption results were more accurately represented by the PSO kinetic model and the Langmuir isotherm model, indicating that CIP uptake was mainly associated with interactions between pollutant molecules and the available surface sites, together with monolayer adsorption behavior. The enhanced adsorption capacity of the MnO_2_/PL biochar nanocomposite is attributed to the synergistic contribution of the porous carbon framework and MnO_2_ nanoparticles, which increased surface functionality, improved site accessibility, and facilitated stronger interactions with CIP molecules. Overall, the developed MnO_2_/PL biochar nanocomposite offers a sustainable, economical, and environmentally benign adsorbent for the removal of pharmaceutical contaminants from aqueous systems. A limitation of this study is the lack of Transmission Electron Microscopy (TEM) analysis, which could provide further evidence of the nanoscale morphology and distribution of MnO_2_ on the biochar. Future work should include TEM and complementary high-resolution techniques, as well as further assessment of long-term structural stability and performance under real wastewater conditions.

## Figures and Tables

**Table 1:** Textural properties and pore characteristics of raw PL, biochar, and MnO_2_/PL biochar.

Sample	BET Surface Area (m^2^/g)	Total Pore Volume (cm^3^/g)	Average Pore Diameter (nm)
PL	0.46	0.002	48
Biochar	1.74	0.012	46
MnO_2_/PL biochar	71	0.136	11

**Table 2:** Kinetic model constants and corresponding correlation coefficients.

Initial CIP Con. (mg/L)	PFO Model		PSO Model		Elovich Model		
	*k*_1_ (min^−1^)	*R* ^2^	*k*_2_ (g/mg min)	*R* ^2^	α (mg/g min)	β (g/mg)	*R* ^2^
52	0.0009	0.368	0.138	0.999	111.7	2.88	0.903

**Table 3:** Isotherm model parameters for CIP adsorption onto MnO_2_/PL biochar at 298 K.

Model	Parmeter	*R* ^2^	RMSE	*χ* ^2^
Langmuir	*Q*_max_ = 24.07 mg/g, *b* = 0.983 L/mg	0.997	1.963	1.520
Freundlich	*K_F_* = 9.88 mg/g (L/mg) ^1/^*^n^*, *n* = 2.874	0.759	4.845	8.829

**Table 4:** Comparison of CIP adsorption capacities derived from the Langmuir isotherm model.

Adsorbent Type	*Q*_max_ (mg/g)	Reference
Bamboo Sawdust Biochar	78.4	[2]
Herbal Residue Biochar	43.7	[29]
Biochar/LaFe_2_O_4_ Nanocomposite	36.5	[30]
CoFe_2_O_4_/Mn–Fe LDH-functionalized pine cone biochar	20.5	[9]
Banana peel biochar	21.0	[6]
Manganese oxide-functionalized magnetic biochar composite	8.4	[31]
Na_2_SiO_3_ -activated carbon from waste bamboo biomass	17.1	[32]
MnO_2_/PL Biochar Nanocomposite	24.1	This study

## Data Availability

The raw data supporting the conclusions of this article will be made available by the authors on request.
